# Cancer Cell Targeting With Functionalized Quantum Dot-Encoded Polyelectrolyte Microcapsules

**DOI:** 10.3389/fchem.2019.00034

**Published:** 2019-01-30

**Authors:** Galina Nifontova, Fernanda Ramos-Gomes, Maria Baryshnikova, Frauke Alves, Igor Nabiev, Alyona Sukhanova

**Affiliations:** ^1^Laboratory of Nano-Bioengineering, Moscow Engineering Physics Institute, National Research Nuclear University MEPhI, Moscow, Russia; ^2^Translational Molecular Imaging, Max-Planck-Institute of Experimental Medicine, Göttingen, Germany; ^3^N. N. Blokhin National Medical Research Center of Oncology, Ministry of Health of the Russian Federation, Institute of Experimental Diagnostic and Biotherapy, Moscow, Russia; ^4^Clinic of Haematology and Medical Oncology, Institute of Diagnostic and Interventional Radiology, University Medical Center Göttingen, Göttingen, Germany; ^5^Laboratoire de Recherche en Nanosciences (LRN-EA4682), Université de Reims Champagne-Ardenne, Reims, France

**Keywords:** quantum dots, polyelectrolyte microcapsules, monoclonal antibody, cytotoxicity, cancer cell targeting

## Abstract

Imaging agents and drug carriers are commonly targeted toward cancer cell through functionalization with specific recognition molecules. Quantum dots (QDs) are fluorescent semiconductor nanocrystals whose extraordinary brightness and photostability make them attractive for direct fluorescent labeling of biomolecules or optical encoding of the membranes and cells. Here, we analyse the cytotoxicity of QD-encoded microcapsules, validate an approach to the activation of the microcapsule's surface for further functionalization with monoclonal antibody Trastuzumab, a humanized monoclonal antibody targeting the extracellular domain of the human epidermal growth factor receptor 2 (HER2) and already in clinical use for the treatment of HER2 positive breast cancer. In addition, we characterize the cell-specific targeting activity of the resultant bio-conjugate by immunofluorescence assay (IFA) and real-time analysis of interaction of the conjugates with live HER2 overexpressing human breast cancer cells. We demonstrate, that encapsulation of QDs into the polymer shell using the layer-by-layer deposition method yields highly fluorescent polyelectrolyte microcapsules with a homogeneous size distribution and biocompatibility upon *in vitro* treatment of cancer cells. Carbodiimide surface activation ensures optimal disperse and optical characteristics of the QD-encoded microcapsules before antibody conjugation. The prepared conjugates of the microcapsules with cancer-specific monoclonal antibody targeting HER2 provide sufficiently sensitive and specific antibody-mediated binding of the microcapsules with live cancer cells, which demonstrated their potential as prospective cancer cell–targeting agents.

## Introduction

Targeted micro- and nanoparticle delivery toward tumors and cancer cells is one of the major research trends in the engineering of anti-cancer theranostic and bioimaging agents. Their controlled transportation and release of their content make it possible to reduce and mitigate off-target effects, ensuring smart delivery of drugs, and diagnostic tools (Fay and Scott, 2011; Wanakule and Roy, 2012). Polyelectrolyte microcapsules loaded with anti-cancer drugs, fluorescent dyes, and metal nanoparticles, e.g., magnetic, plasmonic, or fluorescent semiconductor nanoparticles (quantum dots, QDs), offer a promising opportunity for cancer detection and treatment (Gaponik et al., 2004; Zhao et al., 2006; Vergaro et al., 2011).

Fluorescently labeled polyelectrolyte microcapsules are widely used for imaging of microcapsule transport and release *in vitro* and *in vivo*. The use of QDs as an alternative to conventional, commonly used organic dyes (FITC, TRITC, RITC, etc.) for fluorescent labeling of polyelectrolyte microcapsules results in the formation of highly fluorescent microparticles ensuring their use as promising imaging tools (Gaponik et al., 2003; De Koker et al., 2007; Gao et al., 2016). Quantum dots (QD) attractiveness is explained by their photostability and fluorescent characteristics, including a quantum yield close to 100%, broad absorption spectra, and size-tunable, narrow emission spectra (Bilan et al., 2016). Nevertheless, the chemical composition, size, and surface charge of the semiconductor nanoparticles could limit *in vivo* use of QDs because of their potential toxicity. Quantum dots (QD) encapsulation into the polymer shell of polyelectrolyte microcapsules is a possible way to prevent the QD toxicity for live cell and improve their biocompatibility (Romoser et al., 2011).

Modification of polyelectrolyte microcapsule surface with the biomarkers recognition molecules, such as antibodies (Abs), ensures their specific and selective interaction with the target cells via specific receptors and subsequent internalization. Monoclonal Abs (mAbs) that are conventional immunoglobulin G (IgG) molecules (Mw ~150 kDa) are widely used as specific ligands for bio-functionalization (Johnston et al., 2012). Furthermore, the nano- and microparticles functionalized with Abs are also known to be more biocompatible than non-conjugated ones (Carter et al., 2016).

The polyelectrolyte microcapsule surface can be bio-functionalized with specific Abs by means of passive adsorption or covalent coupling via a chemical crosslinker or by use of additional protein linker, e.g., streptavidin, protein G, or protein A (Deo et al., 2014; Kolesnikova et al., 2017). Covalent coupling appears to be a more advantageous technique for Ab–microparticle conjugation, because the stronger and more effective binding compared to passive adsorption allows controlling the orientation of Ab molecules on the particle surface and maintaining particle colloidal stability, thus ensuring preservation of the Ab and particle functional characteristics.

The goals of the current study were to prepare QD-encoded microcapsules, to estimate their *in vitro* biocompatibility and to develop an approach to bio-functionalization of the QD-encoded microcapsules with mAbs, Trastuzumab, targeting the extracellular domain of the human epidermal growth factor receptor 2 (HER2) and determine whether the designed conjugates specifically interact with cancer cells. SK-BR-3 human breast adenocarcinoma and BT-474 human breast ductal carcinoma cell lines overexpressing HER2 receptor were used as the models. The data can pave the way to further development and investigation of targeted theranostic agents based on QD-encoded polyelectrolyte microcapsules.

## Materials and Methods

### Materials

Poly(allylamine hydrochloride) (PAH) with Mw ~15,000, poly(sodium 4-styrenesulfonate) (PSS) with Mw ~70,000, and polyacrylic acid (PAA) with Mw ~15,000, and bovine serum albumin (BSA) were purchased from Sigma-Aldrich, USA. Sodium carbonate, calcium chloride, and ethylenediaminetetraacetic acid disodium salt dehydrate (EDTA) were obtained from Sigma-Aldrich, Germany. Carboxyl- and sulfhydryl-terminated derivative of 12-unit polyethyleneglycol CT(PEG)_12_, N-hydroxysulfosuccinimide (sulfo-NHS), 1-ethyl-3-(3-dimethylaminopropyl)-carbodiimide hydrochloride (EDC), sulfosuccinimidyl and 4-(N-maleimidomethyl)cyclohexane-1-carboxylate (sulfo-SMCC), 3-[4,5-dimethylthiazol-2-yl]-2,5-diphenyltetrazolium bromide (MTT) were purchased from Thermo Fisher Scientific, USA. CdSe/ZnS (core/shell) QDs with a fluorescence maximum at 590 nm were kindly provided by Dr. P. Samokhvalov (LNBE, MEPhI, Moscow, Russia). The humanized monoclonal anti-HER2 antibody, Trastuzumab (Herceptin^®^), were obtained from Roche, Switzerland.

All the other reagents were of analytical grade and obtained from Sigma-Aldrich, USA. All working polymer and buffer solutions were prepared using MilliQ water (18.2 mΩ·cm) obtained by means of a Direct-Q water purification system (Millipore, France) and additionally filtered through sterile filters with a pore size of 0.22 μm (Millipore, France).

### Preparation of QD-Encoded Microcapsules

Carboxylated PEGylated QDs and QD-encoded microcapsules were prepared as described earlier (Bilan et al., 2017a,b; Nifontova et al., 2018). The technology of microcapsule preparation and QD encoding was based on layer-by-layer deposition of oppositely charged polymers and water-soluble negatively charged QDs onto the surface of calcium carbonate microparticles used as templates. The shell of QD-encoded microcapsules was formed according to the following scheme: CaCO_3_/PAH/PSS/PAH/PSS/PAH/QDs/PAH/PSS/PAH/PSS/PAH/PAA.

Briefly, 0.5 mL of a 2 mg/mL PAH solution in 0.5 M NaCl was added to 0.5 mL of a suspension containing ~3.5 × 10^8^ calcium carbonate microparticles. The suspension was sonicated in an ultrasound bath for a short time and incubated while shaking for 20 min. The excess polymer was washed thrice by centrifugation using ultrapure water. The resultant pellet of PAH-coated calcium carbonate microbeads was resuspended in 0.5 mL of ultrapure water. The next, PSS layer was deposed using 0.5 mL of a 2 mg/mL PSS solution in 0.5 M NaCl under the same conditions. This procedure was repeated to sequentially apply the next PAH, PSS, and PAH layers. After deposition of each polymer layer, the microparticles were washed thrice and resuspended in ultrapure water. CT(PEG)_12_-solubilized CdSe/ZnS QDs characterized by a fluorescence peak at a wavelength of 590 nm, ζ-potential of −29.6 ± 0.8 mV, and a hydrodynamic diameter from 19.2 to 24.3 nm were adsorbed during 80 min of incubation while permanently shaking. Size distribution of the water-soluble QDs is demonstrated by the Figure S1 presented in the Supplementary Materials. Then, the five final layers of oppositely charged PSS and PAH and the sixth one consisting of PAA were applied. Hollow polyelectrolyte microcapsules encoded with QDs were prepared by dissolving the calcium carbonate cores with 0.2 M EDTA (pH 6.5). BSA-coated microcapsules encoded with QDs were obtained by incubation of the microparticles in 10 mM PBS (pH 7.4) containing 1% BSA.

### Chemical Activation of QD-Encoded Microcapsule Surface

Before coupling with the Abs, the surface of the prepared QD-encoded microcapsules was activated by using two approaches based on maleiimide or carbodiimide reaction. Activation of the surface through maleiimide reaction was performed using 1.8 × 10^7^ hollow QD-encoded microcapsules containing the outer layer of PAH in 500 μL of ultrapure water as dispersion medium. Before the reaction, the microcapsules were twice washed by centrifugation (3 min, 9,491 g) with 2 mL of 50 mM phosphate buffer (pH 7.2). Finally, the pellet of microcapsules was resuspended in 955, 977, and 991 μL of 50 mM phosphate buffer (pH 7.2) by vortex and 30-s sonication. Then, 45, 23, and 9 μL of 4.8 mg/mL sulfo-SMCC aqueous solution were added successively. The sulfo-SMCC cross-linker was added in the amounts corresponding to 50×-, 25×-, and 10×-fold molar excesses of the crosslinker over NH_2_ groups on the microcapsule surface. The suspension was gently vortexed and incubated upon permanent shaking at room temperature for 1 h in the dark. After the incubation, microcapsules were thrice washed by centrifugation (3 min, 9,491 g) with 2 mL of 50 mM phosphate buffer (pH 7.2). Then, the pellet was resuspended in 0.5 mL of 50 mM phosphate buffer (pH 7.2), vortexed, and sonicated for 30 s.

Activation of the surface of QD-encoded microcapsules using the carbodiimide reaction was performed according to the standard protocol described earlier (Dunbar and Hoffmeyer, 2013; Brazhnik et al., 2015) using hollow QD-encoded microcapsules containing PAA as the outer layer. Before conjugation, 0.5 mL of the suspension of the microcapsules containing 6 × 10^6^ microparticles and ultrapure water as a dispersion medium was vortexed and subsequently sonicated using an ultrasound bath for 30 s. The suspension was centrifuged for 3 min at 9,491 g. The supernatant was collected, and then the pellet was redispersed in 100 μL of ultrapure water. Afterwards, the suspension was centrifuged under the above conditions, and the supernatant was replaced with 80 μL of 100 mM phosphate buffer (pH 6.2).

Ten microliter of 50 mg/mL sulfo-NHS was added to the suspension of microcapsules and gently vortexed. Subsequently, 10 μL of 50 mg/mL EDC was added to the pre-activated microcapsules, and suspension was gently mixed while shaking using a rotary shaker for 20 min. The activated microcapsules were washed thrice by centrifugation with 250 μl of 50 mM phosphate buffer (pH 7.2). After the final centrifugation, the pellet of microcapsules was resuspended in 0.5 mL of 50 mM phosphate buffer (pH 7.2), vortexed, and sonicated for 30 s.

### Conjugation of QD-Encoded Microcapsules With Monoclonal Antibody

The Ab molecules were conjugated with hollow QD-encoded microcapsules containing PAA as the outer layer by means of the carbodiimide reaction. Before conjugation, 6 × 10^6^ microparticles were dispersed in 0.5 mL of ultrapure water. The microcapsule surface activation using sulfo-NHS and EDC was performed as described above.

The activated microcapsules were washed thrice by centrifugation with 250 μl of 50 mM phosphate buffer (pH 7.2). After final centrifugation, the pellet of microcapsules was vortexed and sonicated for 30 s, and then 3, 30, or 300 μg of monoclonal anti-HER2 antibodies was added. The volume of the reaction mixture was adjusted to 0.5 mL with 50 mM phosphate buffer (pH 7.2). The suspension was permanently mixed while shaking by means of a rotary shaker for 2 h at room temperature in the dark. After the conjugation, the microcapsules were washed by centrifugation with 10 mM PBS (pH 7.4) once and resuspended in 10 mM PBS (pH 7.4) containing 1% of BSA. Afterwards, the suspension of conjugated microcapsules was incubated for 12 h at 4°C. The prepared conjugates were stored in the dark at +4°C. The conjugates were washed of excess BSA with 10 mM PBS (pH 7.4) three times immediately before use.

### Cytotoxicity Study

The cytotoxicity study was performed using SK-BR-3 human breast carcinoma cells (ATCC^®^ HTB-30™). SK-BR-3 cells were cultivated in RMPI-1640 cell culture medium containing 10% of fetal calf serum, 10 mM HEPES (Sigma-Aldrich, USA), 2 mM L-glutamine (Sigma-Aldrich, USA), 40 ng/mL gentamycin (ICN, USA), amino acids, sodium pyruvate, and vitamins (Paneco, Russia) at 37°C in an atmosphere of 5% CO_2_. The cells were kept at the exponential growth phase by subcultivation every 3 or 4 days. Versene solution was used for detachment of the cells attached to the substrate.

For cytotoxicity study, SK-BR-3 cells were seeded into 96-well plates (Costar, USA) containing 180 μL of RMPI-1640 medium. After 24 h of growth, 50–80% of the cell monolayer was formed (~5 × 10^4^ cells per well). The study was performed using BSA-coated QD-encoded and placebo (non-encoded) microcapsules of similar structure and size distribution. Besides, the placebo microcapsules structure was consistent to that of QD-encoded microcapsules: CaCO_3_ (dissolved)/PAH/PSS/PAH/PSS/PAH/PSS/PAH/PSS/PAH/PSS/PAH/PAA. The concentrations of the QD-encoded and placebo microcapsules were determined using KOVA cell chamber system (Fisher Scientific, USA) by counting number of particles per cell grid in the field of view using optical microscopy. The cells were treated with 5 × 10^2^ to 5 × 10^6^ BSA-coated microcapsules encoded with QDs and placebo BSA-coated microcapsules (serving as a control) by incubation in the medium containing the QDs for 24, 48, and 72 h at 37°C in an atmosphere of 5% CO_2_. After each incubation time point, the cells were washed of the microcapsules using 50 mM phosphate buffer (pH 7.4).

Cells viability was evaluated by measuring the mitochondrial dehydrogenase activity. Twenty microliters of 5 mg/ml MTT was added into the wells, and the cells were incubated for 4 h at 37°C in an atmosphere of 5% CO_2_. Mitochondrial dehydrogenases of living cells cleave the tetrazolium ring of MTT, which results in the formation of purple formazan crystals. After formazan crystals were formed, the supernatant was removed, and formazan was dissolved in 150 μL of dimethyl sulfoxide. Then, the plates were thermostated at 37°C and shaken for simultaneous dissolution of formazan crystals. The light absorption by formazan at the wavelength of 540 nm was measured. The cell viability was calculated as the percentage ratio of the absorbance of the cell treated with QD-encoded microcapsules to that of the control cells.

### Functional Activity of the QD-Encoded Microcapsules Conjugated With Antibody

SK-BR-3 cells were seeded into poly-(l-lysine) covered 96-well flat-bottom black, clear-bottom polystyrene plates (Thermo Scientific, USA) containing 100 μL of the RMPI-1640 medium per well. After 24 h, 50–80% of a cell monolayer was formed (~5 × 10^4^ cells per well), the cells were fixed for 15 min by permanently stirring in 100 μL of 10 mM PBS (pH 7.4) containing 4% of paraformaldehyde. After that, the cells were washed thrice with 300 μL of 10 mM PBS (pH 7.4). Then, 300 μL of a blocking agent, 10 mM PBS (pH 7.4) containing 3% casein, were added to each well. The plate was incubated for 2 h while permanently stirring at room temperature. Afterwards, the blocking agent was removed, and 100-μL aliquots containing from 2 × 10^4^ to 1 × 10^6^ QD-encoded microcapsules conjugated with monoclonal anti-HER2 antibodies were added into the wells. The plate was incubated at +4°C overnight in the dark.

After incubation, the cells were thrice washed with 300 μL of 10 mM PBS (pH 7.4). The fluorescence intensity of QD-encoded microcapsule conjugates was analyzed using an Infinite 200 PRO multimodal plate reader (TECAN, Switzerland). The fluorescence intensity was analyzed at an excitation wavelength of 480 nm and emission wavelength of 590 nm. After incubation, the interaction between conjugates of QD-encoded microcapsules and SK-BR-3 cells was also analyzed using optical and fluorescence microscopy.

### Fluorescence Microscopy

The morphology and size distribution of QD-encoded microcapsules with activated surface were analyzed using optical microscopy. The samples were prepared using a 20% aqueous solution of glycerol as a slide mounting media. The plates containing SK-BR-3 and conjugates of QD-encoded microcapsules with antibodies were analyzed without additional plate treatment in the brightfield and fluorescence contrast modes.

The analysis was performed by means of an Axio Observer three microscope (Carl Zeiss, Germany) equipped with an HBO 100 mercury illuminator (Burner Mercury). Fluorescence microscopy was performed using an XF115-2 FITC longpass filter set, including a 505DRLP dichroic filter, a 475AF40 excitation filter, and a 510ALP emission filter (Omega Optical, USA), an LD A-Plan 40x/0.55 M27 lens, an EC Plan-Neofluar100x/1.30 Oil Iris M27 numerical aperture (WD = 0.20 mm) adjustable from 0.7 to 1.3, and Immersol 518F immersion oil (Carl Zeiss, Germany). The obtained images were processed and analyzed for size distribution using the Zen (Carl Zeiss, Germany) and Image J 1.48 v (USA) software.

### Real-Time Live Cancer Cells Targeting With Functionalized QD-Encoded Microcapsules

5 × 10^4^ BT-474 human breast ductal carcinoma cells (ATCC^®^ HTB-20™) were seeded in 24-well plate (Greiner, Germany) and cultured overnight in RMPI-1640 medium. QD-encoded microcapsules conjugated with 3, 30, and 300 μg of antibodies, as well as QD-encoded microcapsules without protein coating were previously diluted in the supplemented cell line medium to 2.5 × 10^6^ and added to the cells. The total working volume per well was 1.5 mL. Afterwards, the plates were placed into the IncuCyte Zoom (Essen BioScience, UK) and imaged in every 2 h. Four fields of view were taken per well regimen at phase contrast and at the green fluorescence excitation filter between 440 and 480 nm, and the emission was acquired using the filters 504–544 nm. Data were analyzed using IncuCyte Zoom software (Essen BioScience, UK) and Fiji (Image processing package based on ImageJ, USA).

### Statistical Analysis

The MS Office Excel 2007 and Origin Pro 2015 software packages were used for statistical analysis of the data. The results are presented as the means and standard deviations for three independent experiments, if not indicated otherwise.

## Results and Discussion

### Cytotoxicity Study

**Figure 1:**
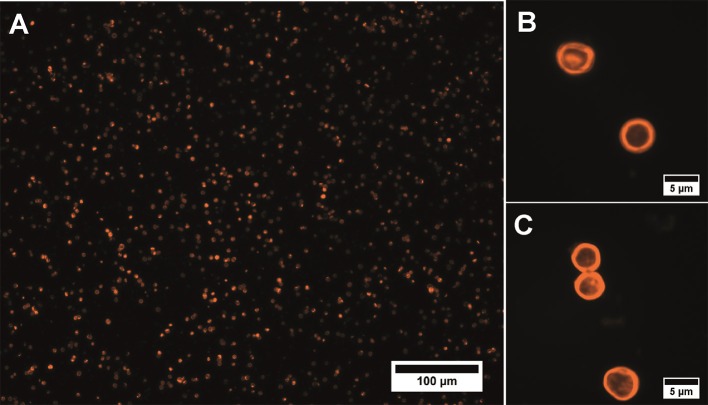
Fluorescent microphotographs of the QD-encoded microparticles at magnifications of **(A)** 20× and **(B,C)** 100×.

The suggested modified technique of polyelectrolyte polymer and QD layer-by-layer deposition onto the surface of calcium carbonate microbeads used as a template and its subsequent removal yielded hollow spherical microparticles with a narrow size distribution and an average size varying from 4.2 to 6.1 μm (Figure 1). The average size of the placebo microcapsules was similar and varied from 4.5 to 6.3 μm. QD-encoded polyelectrolyte microcapsules were characterized by the fluorescent properties that make them promising imaging agents, as noted earlier (Nifontova et al., 2018).

Despite the appropriate fluorescent and disperse characteristics, the presence of QDs in the polymer shell may result in toxicity of the QD-encoded microcapsules for living cells, which is mainly determined by QD physical and chemical properties, such as their size, shape, concentration, and functional groups of the ligand attached to the nanoparticle surface (Win et al., 2013; Sukhanova et al., 2018). Encapsulation of QDs into the polymeric shell consisting of an interpolymeric complex of oppositely charged polymers limits direct interaction of QDs with living cells and seems to be an advantageous approach to diminishing the adverse effects of QDs *in vivo* (Romoser et al., 2011; Bazylinska et al., 2016).

Hence, we analyzed SK-BR-3 cell viability after 24–72 h of the cell incubation with QD-encoded microcapsules and QD-free (placebo) microcapsules. SK-BR-3 cells were used as an *in vitro* model to evaluate the possible cytotoxic effect of the QD-encoded microcapsules, which represent the basis for further development of the theranostic system specific for HER2-overexpressing tissues, especially breast tumors.

**Figure 2:**
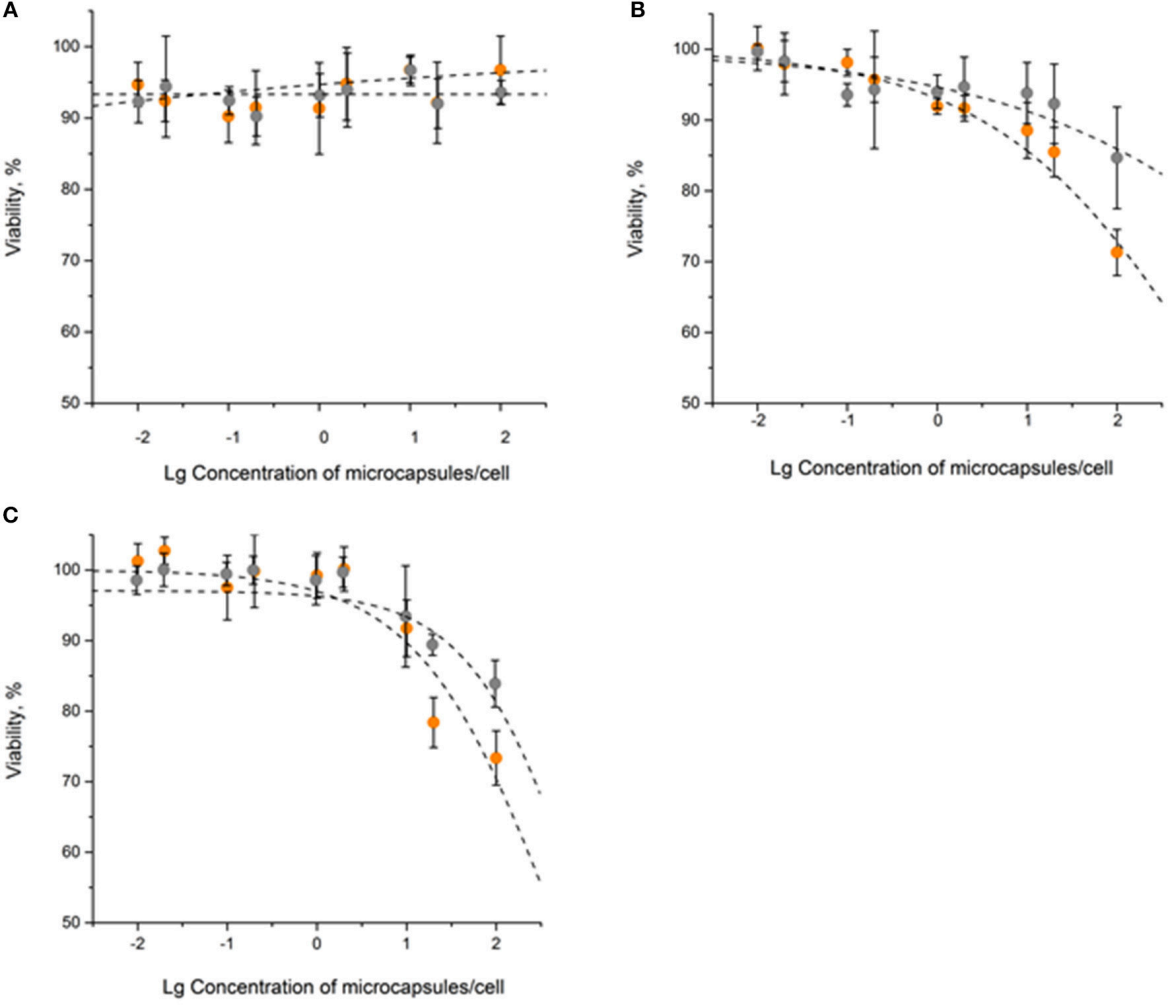
The *in vitro* cytotoxicity of the QD-encoded microcapsules (orange circles) and placebo microcapsules (gray circles): **(A)** 24 h, **(B)** 48 h, **(C)** 72 h.

The obtained data showed only a minor effect of QD-encoded microcapsules on the SK-BR-3 cell viability: the 20% cell viability decrease was observed only after 48 and 72 h of cells incubation with the QD-encoded microcapsules (Figure 2). Hence, the IC_20_ values were calculated to determine a possible range of microcapsule working concentrations providing cell viability more than 80% what was necessary to perform further living cell studies.

After 24 h of the SK-BR-3 cells incubation with the QD-encoded and QD-free microcapsules, no significant cell viability decrease was observed, so that it was impossible to estimate the IC_20_ (Figure 2A and Table 1). Longer exposures to the QD-encoded microcapsules, for 48 and 72 h, resulted in a decrease in cell viability to similar degrees (Figures 2B,C), without a significant difference between the corresponding IC_20_ values (Table 1). Exposure to the placebo microcapsules for 48 or 72 h only negligibly affected cell viability. In the case of placebo microcapsules, there was no 20% cell mortality after any period of incubation; hence, polyelectrolyte microcapsules *per se* are not cytotoxic. A negligible decrease in cell viability at a high concentration of microcapsules (100 microcapsules per cell) may have been caused by the formation of microcapsule aggregates on the cell surface after longer incubation time (Shen et al., 2018). QD-encoded microcapsule cytotoxicity for living cells proved to be low and was observed after long incubation time and mainly at high concentrations of the microcapsules. This justifies the investigation of the possibility of their further functionalization with Abs and their targeting toward living cells.

**Table 1:** IC_20_ values of QD-encoded microcapsules.

**Time interval, h**	**IC_**20**_, microcapsules/cell**
24	NA
48	37.4 ± 8.5^*^
72	44.4 ± 7.4^*^

*The results are mean ± SD for three independent measurements*.

* *Non-significant differences between the IC_20_ values (p > 0.05 according to Student's t-test); NA, data not available; IC_20_, the concentration of the QD-encoded microcapsules at which the cell viability is 80%*.

### Microcapsule Surface Activation

The targeting of QD-encoded microcapsules based on functionalization of the microparticle surface with Abs is provided by activation of the microcapsule surface with specific crosslinkers and subsequent protein coupling. Preliminarily, the influence of the heterobifunctional NHS ester–maleimide (sulfo-SMCC) and carbodiimide (EDC in combination with sulfo-NHS) crosslinkers used for surface activation on the microcapsule disperse characteristics was analyzed.

The functional groups of the polymers forming the outer layers of the microcapsule surface are involved in antibody conjugation. Sulfo-SMCC activation results in primary binding of the crosslinker to the PAH-coated surface of the QD-encoded microcapsules and involves subsequent coupling of antibody thiol-containing fragments forming a thioester bond (Hermanson, 2008b).

**Figure 3:**
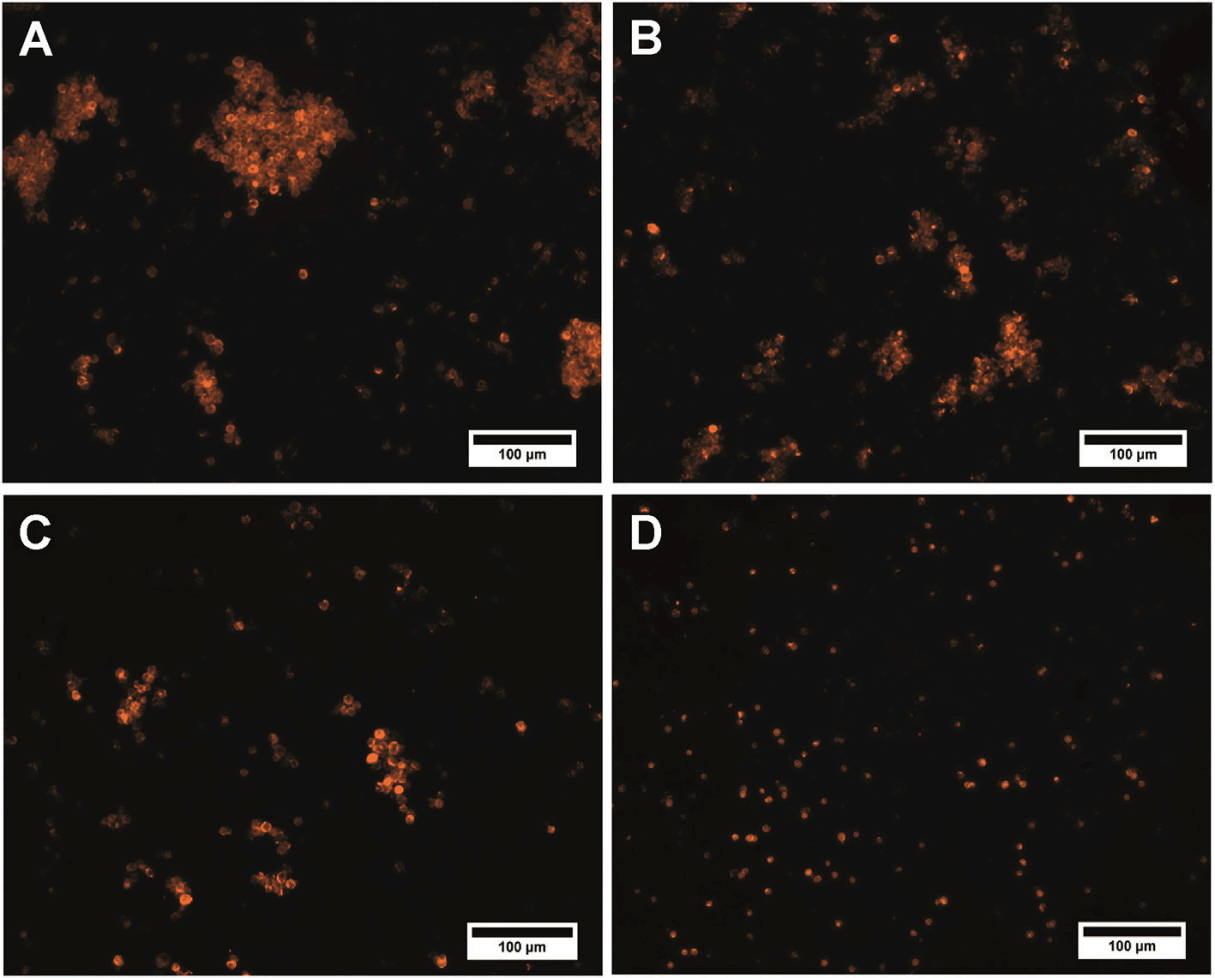
QD-encoded microcapsules (*n* = 300) functionalized with **(A)** a 50× excess of sulfo-SMCC; **(B)** a 25× excess of sulfo-SMCC; **(C)** a 10× excess of sulfo-SMCC; and **(D)** sulfo-NHS and EDC.

Activation of the microcapsule surface via maleimide reactions using sulfo-SMCC resulted in inconvertible conglomeration of the polyelectrolyte microcapsules. The size of the conglomerates and amount of microcapsules constituting them rose as the crosslinker content in the reaction mixture increased (Figures 3A–C). The use of a 50 × sulfo-SMCC excess provides polydispersity of the microcapsule suspension. In this case, both single particles and conglomerates could be observed. Microcapsule conglomerates were characterized by irregular shapes and sizes from 11.0 to 134.3 μm (Figure 3A). However, the use of a 25× and a 10× sulfo-SMCC excesses resulted in a decrease in size (from 10.0 to 135.0 μm and from 10.7 to 125.0 μm) and content of both common and large-sized conglomerates in the reaction mixture, whereas the content of single particles was larger (Figures 3B,C). The observed conglomeration may be explained by interlinking of the maleimide-activated microcapsules, which may have been induced by covalent binding of microcapsules facilitated with potential non-covalent, hydrophobic interaction between the activated microcapsules due to the presence of a cyclohexane fragment in the crosslinker structure. Thus, the use of maleimide activation results in decline of the disperse characteristics of the microparticle suspension at a 50× excess of the crosslinker, as well as upon a 5-fold decrease in the sulfo-SMCC excess, which limits the use of the activation mediated by sulfo-SMCC in the case of the QD-encoded polyelectrolyte microcapsules.

Alternatively, carbodiimide activation by using zero-length crosslinker was studied as an optional way to functionalize the microparticle surface. The exposed carboxyl groups of PAA constituting the outer layer of the polymeric shell of the QD-encoded microcapsules are activated with EDC in the presence of sulfo-NHS, yielding a sulfo-NHS–ester intermediate structure on the surface of the microcapsules. Addition of sulfo-NHS is known to stabilize the O-acylisourea intermediate by forming a succinimide ester, which increases the activation efficiency (Thanh and Green, 2010). Primary NH_2_ groups of the antibodies are supposed to bind to the pre-activated surface of the microcapsules due to the formation of a covalent amine bond (Hermanson, 2008a).

Analysis of the microcapsule disperse characteristics after carbodiimide reaction revealed the absence of the aggregated microcapsules. The size distribution of the EDC-sulfo-NHS-activated microcapsules is similar to that of the initial microcapsules, with the average size falling in about the same range, from 4.2 to 6.2 μm (Figures 1A, 3D). The differences between the size distributions of the EDC-sulfo-NHS-activated and initial QD-encoded microcapsules were found to be non-significant (one-way ANOVA, *p* > 0.05). Hence, the protocol used for particle surface activation with the use of EDC and sulfo-NHS allows preserving the microcapsule dispersity. The QD-encoded microcapsules with the EDC activated surface can be used for further efficient functionalization of microcapsule with specific recognition molecules.

### QD-Encoded Microcapsules Functionalization With Specific Antibody

Carbodiimide-activated QD-encoded microcapsules were conjugated with the humanized anti-HER2 mAb via primary NH_2_ groups of Ab, providing non-selective and randomly oriented covalent amide bond coupling of Ab (Greene et al., 2017). The ratios between the numbers of 4.6-μm microcapsules and mAb molecules were 1: 2 × 10^6^, 1: 2 × 10^7^, 1: 2 × 10^8^. This ensured, in each case, the molar excess of protein to spherically shaped microparticles with a mean diameter of 4.6 μm that was optimal for the coupling process. After the coupling, the microcapsules were washed of the excess of mAb and their surface was additionally treated with BSA to block the remaining EDC/sulfo-NHS-activated sites free of mAb and to improve biocompatibility of the microcapsules.

### Conjugate Functional Activity

The functional activity of the prepared conjugated microcapsules, defined as the ability of the prepared conjugates to specifically interact with cells, was primary studied using human SK-BR-three cells as a HER2-overexpressing tumor cell based model. To minimize the possibility of non-specific interaction between the cells and microcapsules, the cell surface was first treated with 3% casein and washed of the unbound microparticles before the analysis. The tested concentration range of the conjugated QD-encoded microcapsules was selected according to the cytotoxicity data and did not exceed the 20 microcapsules per cell, the concentration point which was shown to provide no cell toxicity effects as was shown in Figure 2A.

**Figure 4:**
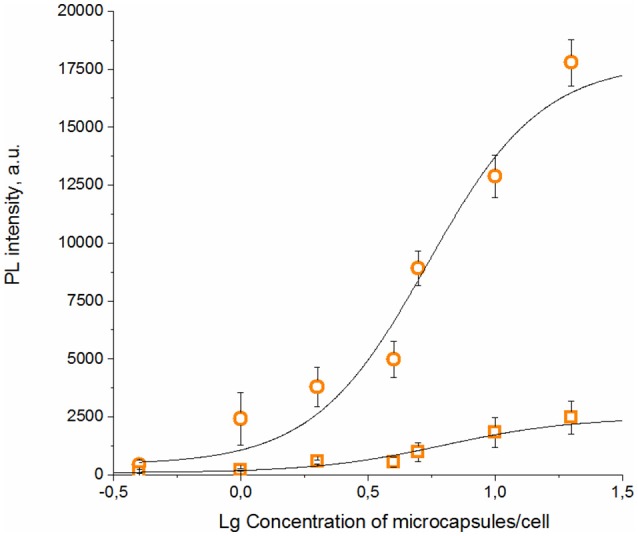
Binding curves of the QD-encoded polyelectrolyte microcapsules conjugated with trastuzumab (circles) and QD-encoded microcapsules without protein coating as a control (squares) are shown.

**Figure 5:**
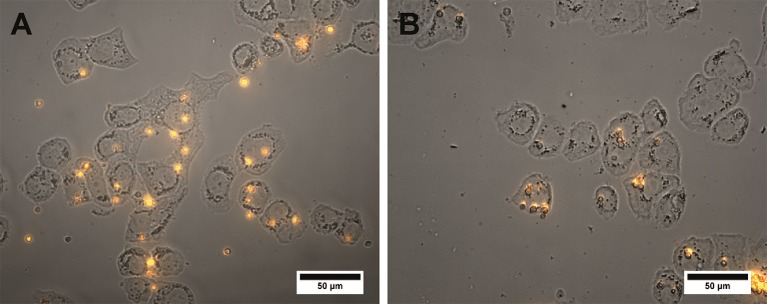
Microphotographs of the SK-BR-3 cells after overnight exposure to the QD-encoded microcapsules conjugated with **(A)** trastuzumab and **(B)** non-conjugated QD-encoded microcapsules are shown. The amount of the microcapsules was 20 per cell. The image represents a merge of a brightfield and fluorescent channels. Image processing was performed using the Image J 1.48 v software.

The specific binding ability of the QD-encoded microcapsule conjugates to SK-BR-3 cells overexpressing HER2 evaluated at the 4°C is shown in Figure 4. The fluorescent signals of non-conjugated microcapsules and microcapsule conjugates with anti HER2 mAb differed significantly (*p* < 0.05, Student's *t*-test), starting from a microparticle-to-cell ratio of 0: 1. However, at the microparticle-to-cell ratios from 10 to 20, a tendency of further increase in the signal intensity in case of the anti HER2 mAb-conjugated microcapsules was observed. The fluorescence intensity signal of the conjugated microcapsules in comparison with those of the microcapsules without protein coating after interaction with casein-blocked SK-BR-3 cell surface could have testified mainly specific binding in case of the conjugated microcapsules. The obtained data are in good accordance with microphotographs of the wells of the plates containing SK-BR-3 cells and QD-encoded microcapsules (Figure 5A), where a higher efficiency of binding of the microcapsules conjugated with mAb in comparison with non-conjugated samples (Figure 5B) were observed. In case of the QD-encoded microcapsules without protein coating, it was shown that, with increasing number of microparticles per cell, the probability of non-specific binding also rises, and a fluorescence signal of < 10% of the total signal was obtained (Figure 4). As seen in Figure 5B, several single microparticles located on the cell surface could be observed at the highest concentration analyzed.

**Figure 6:**
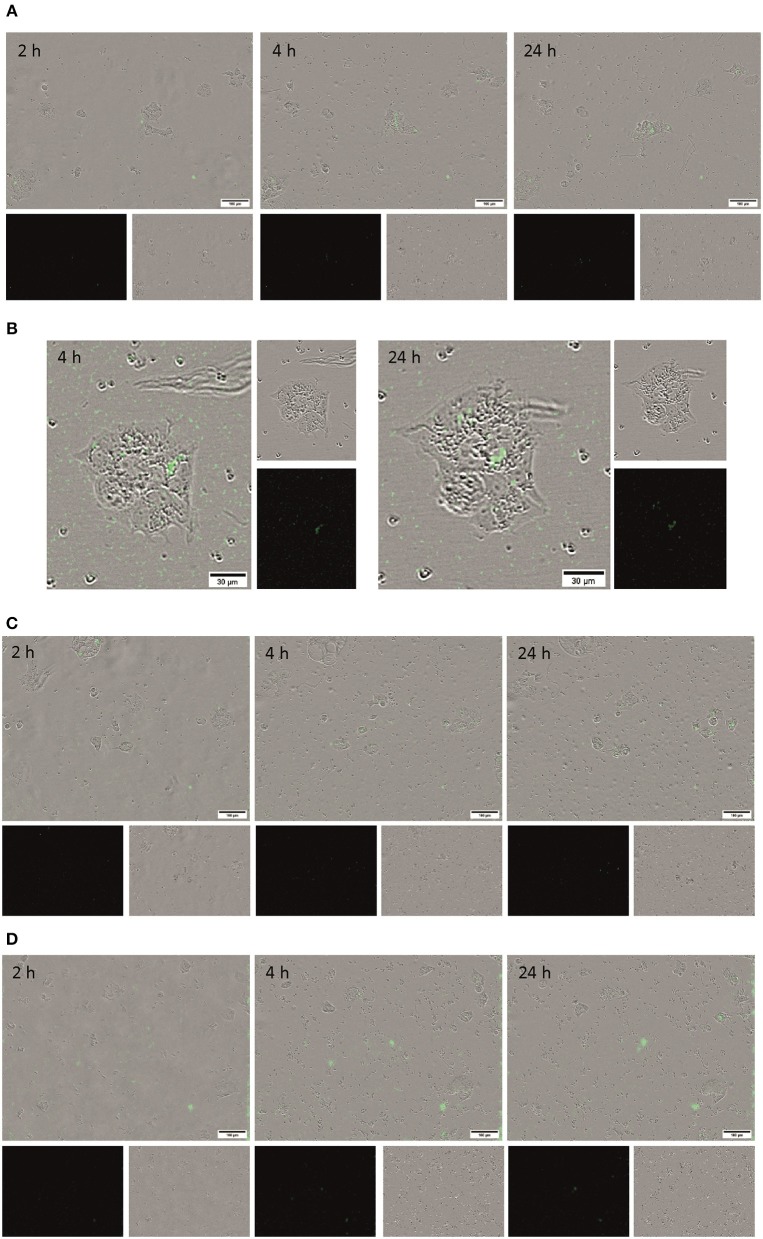
Microphotographs of the living BT-474 cells after 2, 4, and 24 h of exposure to the QD-encoded microcapsules conjugated with **(A)** 3 μg, **(B)** 30 μg, and **(C)** 300 μg of trastuzumab and **(D)** QD-encoded microcapsules without protein coating as control are shown. The loaded amount of the microcapsules was 50 per cell.

Living BT-474 human breast tumor cells as a further HER2-overexpressing cell model in combination with the Incucyte Zoom live-cell analysis equipment were used to follow in real-time the interaction of the conjugated fluorescent microcapsules containing different quantities of anti-HER2 mAbs with living cells. QD-encoded microcapsules without protein coating were used as controls to analyse non-specific binding of the microcapsules to the BT-474 cells. Figures 6A–C show that the quantity of the QD-encoded microcapsules conjugates detectable on the cell surface increases upon the increase of the content of anti-HER2 mAb on the microparticle surface at primary exposition times (2, 4, and 12 h). This fact is also approved by the microphotographs and videos of real-time binding of the conjugates to living cells presented in Supplementary Materials (Videos S1, S2, and S3). Importantly, an amount of the non-conjugated microcapsules detected on the cell surface at the pre-determined time points was found to be negligible what agrees with the immunofluorescence assay data and demonstrates the specific character of binding of the anti-HER2 mAb-containing microcapsules with the cells (Figure 6D).

Anti-HER2 mAb Trastuzumab (Herceptin^®^) used in this study is characterized by high affinity to its target, the extracellular domain of HER-2, with the K_D_ value of ~5 nM (De Lorenzo et al., 2005). The specific binding sites of anti-HER2 mAb conjugated via the zero-length carbodiimide crosslinker are located predominantly at a significant distance from the microparticle surface, what provides it with the ability to interact with the cell plasma membrane-bound HER-2 antigen. However, conjugates obtained by loading 30 and 300 μg of the anti-HER2 mAbs exhibited higher binding capacity to BT-474 cells in comparison with microcapsules conjugated with 3 μg of the anti-HER2 mAbs. This can be explained by higher density of covalently coupled anti-HER2 mAb molecules on the surface of the microcapsules in the case of 30 and 300 μg of anti-HER2 mAb conjugates, respectively, what have resulted in higher quantity of the active binding centers of the anti-HER2 mAbs on the microparticle surface and, hence, more efficient binding of these conjugates to the living cells.

Longer (up to 24 h) exposition of the microcapsules conjugated with 30 and 300 μg of anti-HER2 mAb to living cells resulted in an increase of the quantity of the microcapsule attached to the cell surface for all the microcapsule probes including control non-conjugated ones, what indicated a possible contribution of unspecific interaction with the cells (Figure 6D, the 24 h time-point). However, the quantity of the conjugates on the cell surface was orders of magnitude higher for the microcapsule conjugates in comparison with non-conjugated microcapsules. The obtained data proved the specificity of interaction between microcapsules conjugated with anti-HER2 mAb and demonstrated the efficiency of the use QD-encoded microcapsules containing 30 to 300 μg of the anti-HER2 mAb as potential tumor cell-specific imaging tools and delivery systems to tumor cells.

## Conclusions

Embedment of QDs into the polymer shell of the polyelectrolyte microcapsules according to the suggested encoding procedure ensures a low toxicity for living cells and biocompatibility of the prepared QD-encoded microcapsules. The use of carbodiimide-mediated surface activation of the QD-encoded microcapsules provides preservation of their optical and dispersion characteristics, as well as permit efficient antibody-functionalization. The prepared conjugates of the microcapsules with anti-HER2 mAbs exhibit the capacity for specific interaction with HER2 overexpressing tumor cell models. Hence, QD-encoded microcapsules developed in this study have been shown to be potential biocompatible fluorescent agents for live cell targeting, they may be tagged with different antibodies and serve as the basic platform for further development of a targeted systems for diagnosis and therapy of a variety of tumor entities.

## Author Contributions

GN, IN, and AS designed the study and all the experiments. GN synthesized polyelectrolyte microcapsules encoded with QDs and conjugated them with mAb. MB and GN performed the cytotoxicity assay. MB, GN, FR-G, and FA performed the functional tests and microscopy experiments. GN, IN, and AS prepared the manuscript. All authors discussed the results and commented on the manuscript.

### Conflict of Interest Statement

The authors declare that the research was conducted in the absence of any commercial or financial relationships that could be construed as a potential conflict of interest.

## References

[B1] Bazylinska U. Wawrzynczyk D. Kulbacka J. Frackowiak R. Cichy B. Bednarkiewicz A.. (2016). Polymeric nanocapsules with up-converting nanocrystals cargo make ideal fluorescent bioprobes. Sci. Rep. 6:29746. 10.1038/srep29746 27406954 PMC4942829

[B2] Bilan R. Nabiev I. Sukhanova A. (2016). Quantum dot-based nanotools for bioimaging, diagnostics, and drug delivery. ChemBioChem 17, 2103–2114. 10.1002/cbic.201600357 27535363

[B3] Bilan R. S. Ametzazurra A. Brazhnik K. Escorza S. Fernández D. Uríbarri M.. (2017a). Quantum-dot-based suspension microarray for multiplex detection of lung cancer markers: preclinical validation and comparison with the Luminex xMAP system. Sci. Rep. 7, 1–10. 10.1038/srep44668 28300171 PMC5353738

[B4] Bilan R. S. Krivenkov V. A. Berestovoy M. A. Efimov A. E. Agapov I. I. Samokhvalov P. S.. (2017b). Engineering of optically encoded microbeads with FRET-free spatially separated quantum-dot layers for multiplexed assays. ChemPhysChem 18, 970–979. 10.1002/cphc.201601274 28194871

[B5] Brazhnik K. Sokolova Z. Baryshnikova M. Bilan R. Efimov A. Nabiev I.. (2015). Quantum dot-based lab-on-a-bead system for multiplexed detection of free and total prostate-specific antigens in clinical human serum samples. Nanomed. NBM 11, 1065–1075. 10.1016/j.nano.2015.03.003 25804411

[B6] Carter T. Mulholland P. Chester K. (2016). Antibody-targeted nanoparticles for cancer treatment. Immunotherapy 8, 941–958. 10.2217/imt.16.11 27381686

[B7] De Koker S. De Geest B. G. Cuvelier C. Ferdinande L. Deckers W. Hennink W. E. (2007). *In vivo* cellular uptake, degradation, and biocompatibility of polyelectrolyte microcapsules. Adv. Funct. Mater. 17, 3754–3763. 10.1002/adfm.200700416

[B8] De Lorenzo C. Cozzolino R. Carpentieri A. Pucci P. Laccetti P. D'Alessio G. (2005). Biological properties of a human compact anti-ErbB2 antibody. Carcinogenesis 26, 1890–1895. 10.1093/carcin/bgi146 15930029

[B9] Deo D. I. Gautrot J. E. Sukhorukov G. B. Wang W. (2014). Biofunctionalization of PEGylated microcapsules for exclusive binding to protein substrates. Biomacromolecules 15, 2555–2562. 10.1021/bm500412d 24848418

[B10] Dunbar S. A. Hoffmeyer M. R. (2013). Microsphere-based multiplex immunoassays: development and applications using Luminex® xMAP® technology, in The Immunoassay Handbook. Theory and Applications of Ligand Binding, ELISA and Related Techniques, eds Wild D. John R. Sheehan C. Binder S. He J. (Oxford: Elsevier Ltd), 157–174. 10.1016/B978-0-08-097037-0.00073-7

[B11] Fay F. Scott C. J. (2011). Antibody-targeted nanoparticles for cancer therapy. Immunotherapy 3, 381–394. 10.2217/IMT.11.5 21395380

[B12] Gao H. Sapelkin A. V. Titirici M. M. Sukhorukov G. B. (2016). *In situ* synthesis of fluorescent carbon dots/polyelectrolyte nanocomposite microcapsules with reduced permeability and ultrasound sensitivity. ACS Nano 10, 9608–9615. 10.1021/acsnano.6b05088 27684330

[B13] Gaponik N. Radtchenko I. L. Gerstenberger M. R. Fedutik Y. A. Sukhorukov G. B. Rogach A. L. (2003). Labeling of biocompatible polymer microcapsules with near-infrared emitting nanocrystals. Nano Lett. 3, 369–372. 10.1021/nl0259333

[B14] Gaponik N. Radtchenko I. L. Sukhorukov G. B. Rogach A. L. (2004). Luminescent polymer microcapsules addressable by a magnetic field. Langmuir 20, 1449–1452. 10.1021/la035914o 15803733

[B15] Greene M. K. Richards D. A. Nogueira J. C. F. Campbell K. Smyth P. Fernández M.. (2017). Forming next-generation antibody-nanoparticle conjugates through the oriented installation of non-engineered antibody fragments. Chem. Sci. 9, 79–87. 10.1039/c7sc02747h 29629076 PMC5869316

[B16] Hermanson G. T. (ed.). (2008a). Bioconjugate reagents, in Bioconjugate Techniques (London: Academic Press), 215–222.

[B17] Hermanson G. T. (ed.). (2008b). Bioconjugate techniques, in Bioconjugate Techniques (London: Academic Press), 766–773. 10.1016/B978-0-12-370501-3.00003-5

[B18] Johnston A. P. Kamphuis M. M. Such G. K. Scott A. M. Nice E. C. Heath J. K.. (2012). Targeting cancer cells: controlling the binding and internalization of antibody-functionalized capsules. ACS Nano 6, 6667–6674. 10.1021/nn3010476 22872125

[B19] Kolesnikova T. A. Kiragosyan G. Le T. H. Springer S. Winterhalter M. (2017). Protein A functionalized polyelectrolyte microcapsules as a universal platform for enhanced targeting of cell surface receptors. ACS Appl. Mater. Interfaces 9, 11506–11517. 10.1021/acsami.7b01313 28290659

[B20] Nifontova G. Zvaigzne M. Baryshnikova M. Korostylev E. Ramos-Gomes F. Alves F.. (2018). Next-generation theranostic agents based on polyelectrolyte microcapsules encoded with semiconductor nanocrystals: development and functional characterization. Nanoscale Res. Lett. 13:30. 10.1186/s11671-018-2447-z 29372483 PMC5785454

[B21] Romoser A. Ritter D. Majitha R. Meissner K. E. McShane M. Sayes C. M. (2011). Mitigation of quantum dot cytotoxicity by microencapsulation. PLoS ONE 6:e22079. 10.1371/journal.pone.0022079 21814567 PMC3140988

[B22] Shen H. Li F. Wang D. Yang Z. Yao C. Ye Y. (2018). Chitosan-alginate BSA-gel-capsules for local chemotherapy against drug-resistant breast cancer. Drug Des. Devel. Ther. 2, 921–934. 10.2147/DDDT.S158001 PMC5914552 29719378

[B23] Sukhanova A. Bozrova S. Sokolov P. Berestovoy M. Karaulov A. Nabiev I. (2018). Dependence of nanoparticle toxicity on their physical and chemical properties. Nanoscale Res. Lett. 13:44. 10.1186/s11671-018-2457-x 29417375 PMC5803171

[B24] Thanh N. T. K. Green L. A. W. (2010). Functionalisation of nanoparticles for biomedical applications. Nano Today 5, 213–230. 10.1016/j.nantod.2010.05.003

[B25] Vergaro V. Scarlino F. Bellomo C. Rinaldi R. Vergara D. Maffia M.. (2011). Drug-loaded polyelectrolyte microcapsules for sustained targeting of cancer cells. Adv. Drug Deliv. Rev. 63, 847–864. 10.1016/j.addr.2011.05.007 21620912

[B26] Wanakule P. Roy K. (2012). Disease-responsive drug delivery: the next generation of smart delivery devices. Curr. Drug Metab. 13, 42–49. 10.2174/138920012798356880 22385534

[B27] Win K. Y. Ye E. Teng C. P. Jiang S. Han M. Y. (2013). Engineering polymeric microparticles as theranostic carriers for selective delivery and cancer therapy. Adv. Healthc. Mater. 2, 1571–1575. 10.1002/adhm.201300077 23712912

[B28] Zhao Q. Zhang S. Tong W. Gao C. Shen J. (2006). Polyelectrolyte microcapsules templated on poly (styrene sulfonate)-doped CaCO3 particles for loading and sustained release of daunorubicin and doxorubicin. Eur. Polym. J. 42, 3341–3351. 10.1016/j.eurpolymj.2006.09.005

