# Supplementary material for: Cancer Cell Targeting With Functionalized Quantum Dot-Encoded Polyelectrolyte Microcapsules
Source: Front Chem. 2019 Jan 30;7:34. doi: 10.3389/fchem.2019.00034 (PMC6363708; doi:10.3389/fchem.2019.00034)
Supplement: Supplementary file 1 [file Presentation_1.pptx]

## Slide 1
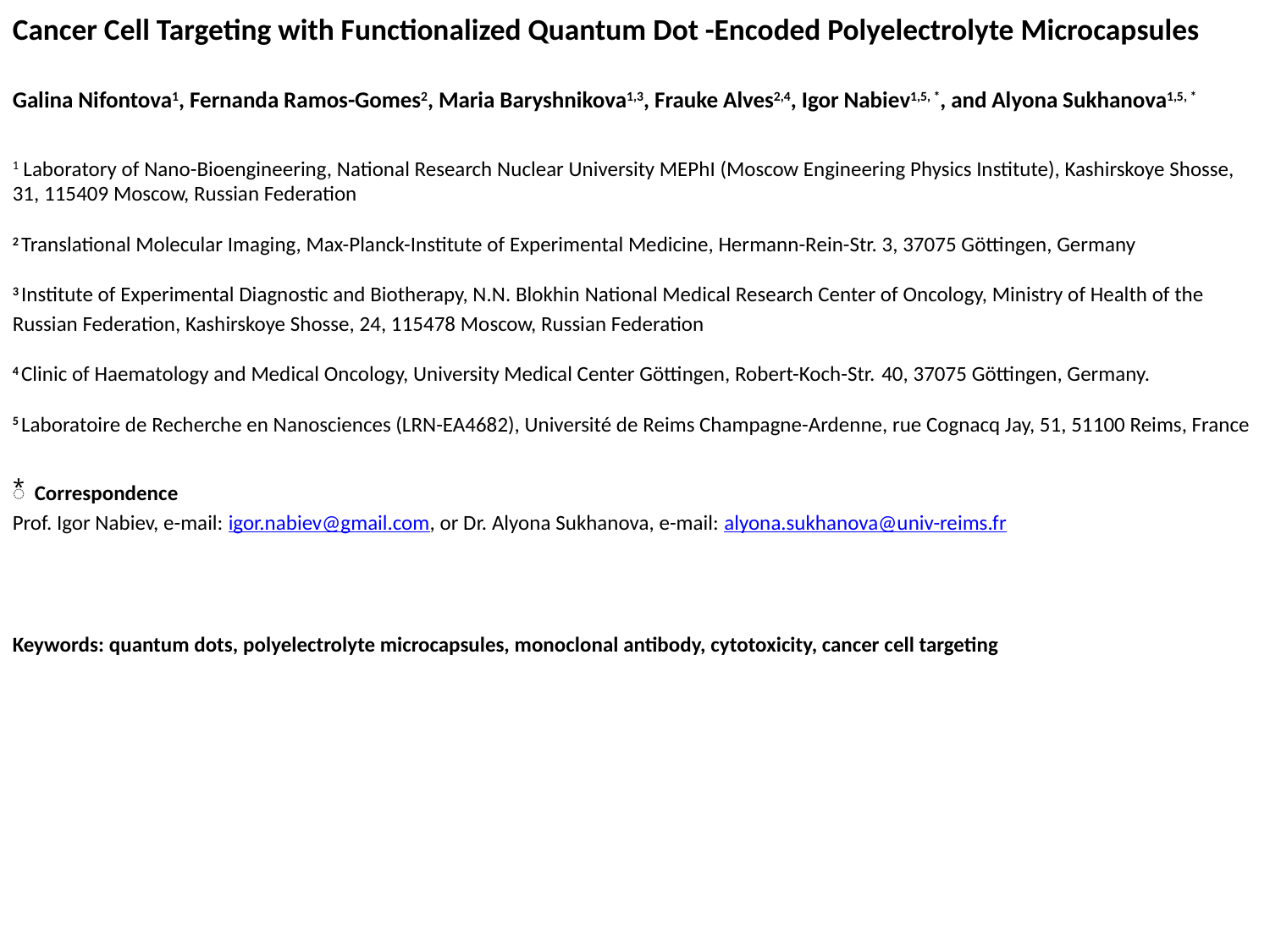

Cancer Cell Targeting with Functionalized Quantum Dot -Encoded Polyelectrolyte Microcapsules
Galina Nifontova1, Fernanda Ramos-Gomes2, Maria Baryshnikova1,3, Frauke Alves2,4, Igor Nabiev1,5, *, and Alyona Sukhanova1,5, *
1 Laboratory of Nano-Bioengineering, National Research Nuclear University MEPhI (Moscow Engineering Physics Institute), Kashirskoye Shosse, 31, 115409 Moscow, Russian Federation
2 Translational Molecular Imaging, Max-Planck-Institute of Experimental Medicine, Hermann-Rein-Str. 3, 37075 Göttingen, Germany
3 Institute of Experimental Diagnostic and Biotherapy, N.N. Blokhin National Medical Research Center of Oncology, Ministry of Health of the Russian Federation, Kashirskoye Shosse, 24, 115478 Moscow, Russian Federation
4 Clinic of Haematology and Medical Oncology, University Medical Center Göttingen, Robert-Koch-Str. 40, 37075 Göttingen, Germany.
5 Laboratoire de Recherche en Nanosciences (LRN-EA4682), Université de Reims Champagne-Ardenne, rue Cognacq Jay, 51, 51100 Reims, France
⃰ Correspondence
Prof. Igor Nabiev, e-mail: igor.nabiev@gmail.com, or Dr. Alyona Sukhanova, e-mail: alyona.sukhanova@univ-reims.fr
Keywords: quantum dots, polyelectrolyte microcapsules, monoclonal antibody, cytotoxicity, cancer cell targeting

## Slide 2
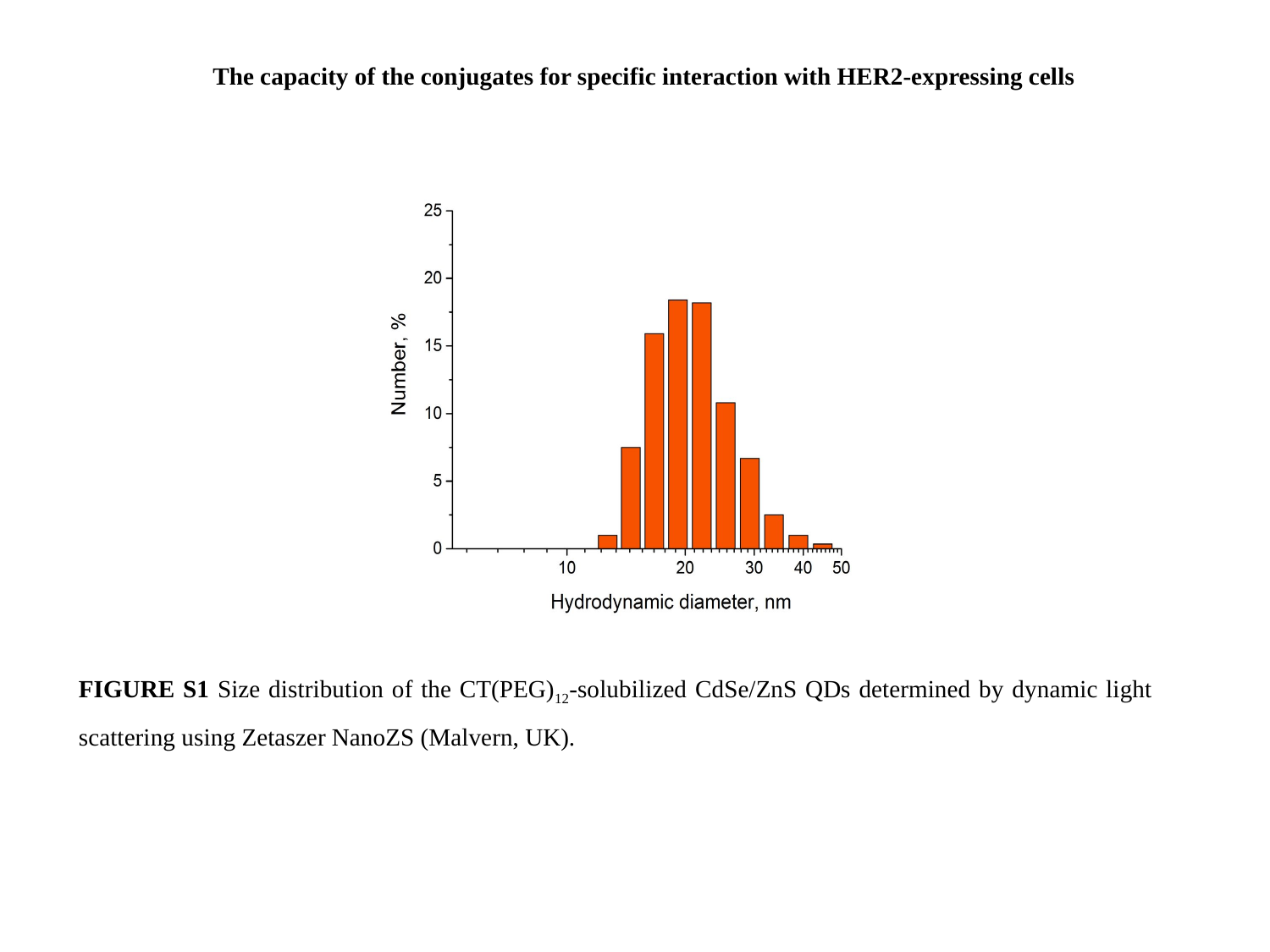

The capacity of the conjugates for specific interaction with HER2-expressing cells
FIGURE S1 Size distribution of the CT(PEG)12-solubilized CdSe/ZnS QDs determined by dynamic light scattering using Zetaszer NanoZS (Malvern, UK).

## Slide 3
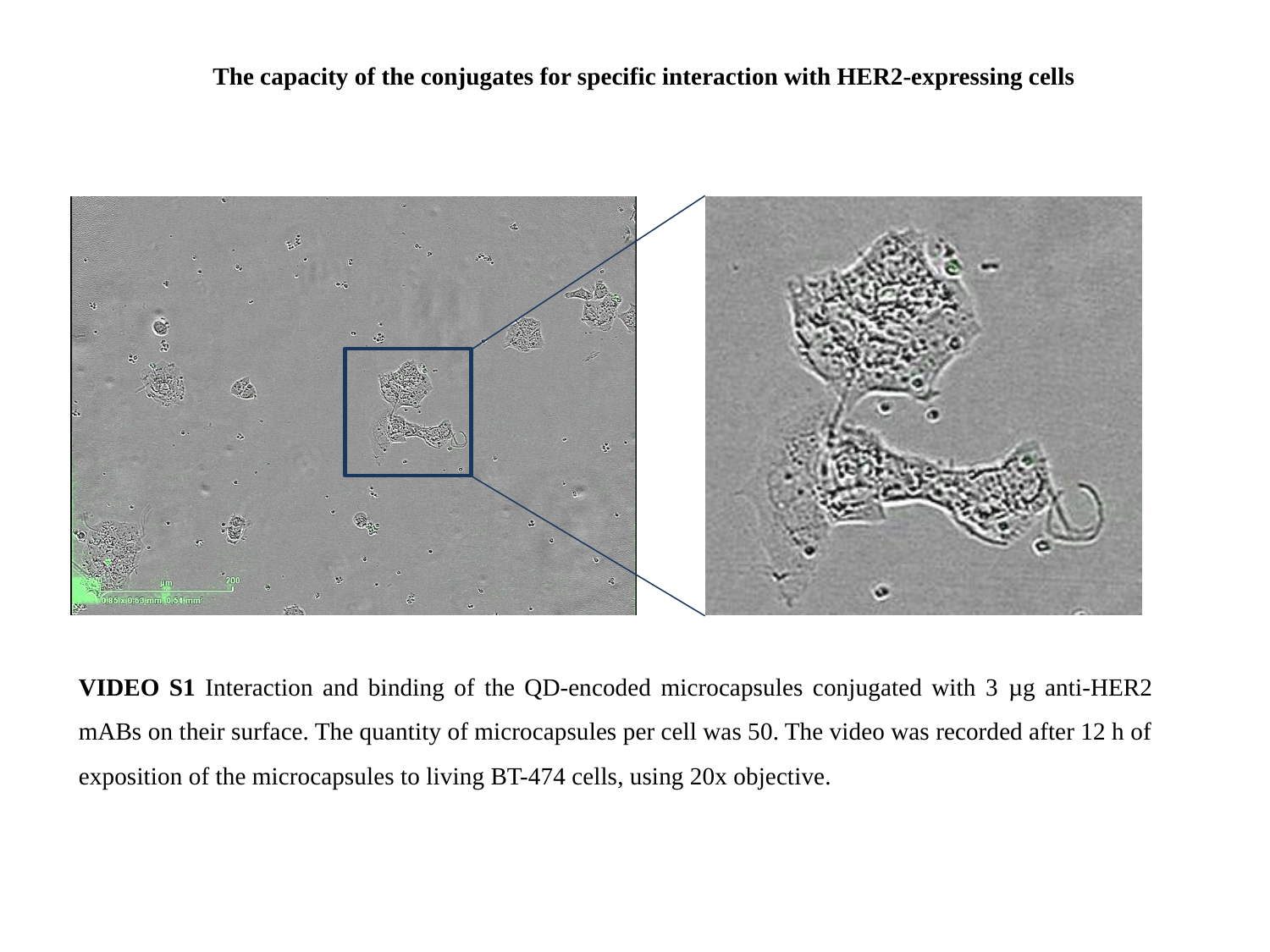

The capacity of the conjugates for specific interaction with HER2-expressing cells
VIDEO S1 Interaction and binding of the QD-encoded microcapsules conjugated with 3 µg anti-HER2 mABs on their surface. The quantity of microcapsules per cell was 50. The video was recorded after 12 h of exposition of the microcapsules to living BT-474 cells, using 20x objective.

## Slide 4
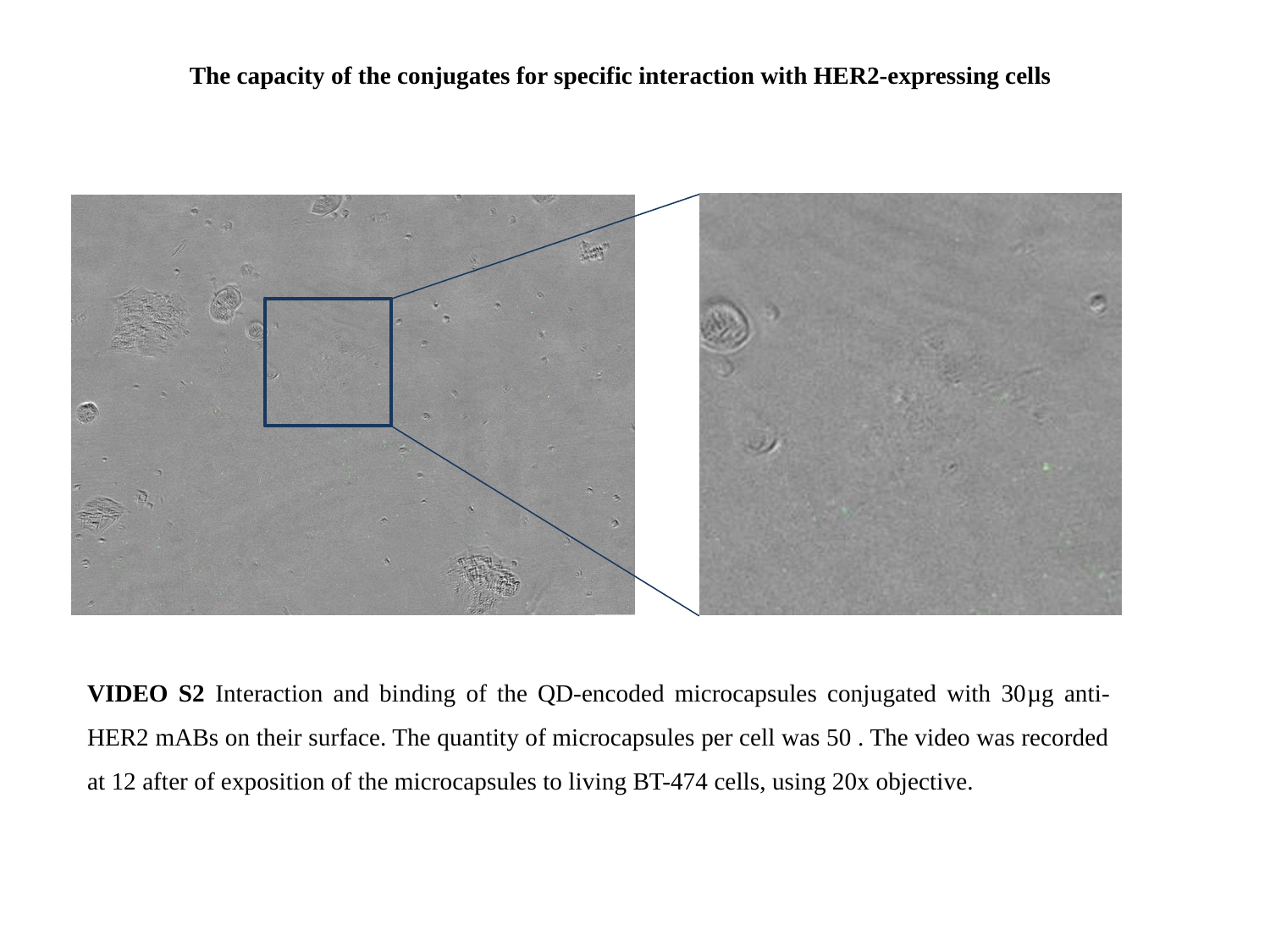

The capacity of the conjugates for specific interaction with HER2-expressing cells
VIDEO S2 Interaction and binding of the QD-encoded microcapsules conjugated with 30µg anti-HER2 mABs on their surface. The quantity of microcapsules per cell was 50 . The video was recorded at 12 after of exposition of the microcapsules to living BT-474 cells, using 20x objective.

## Slide 5
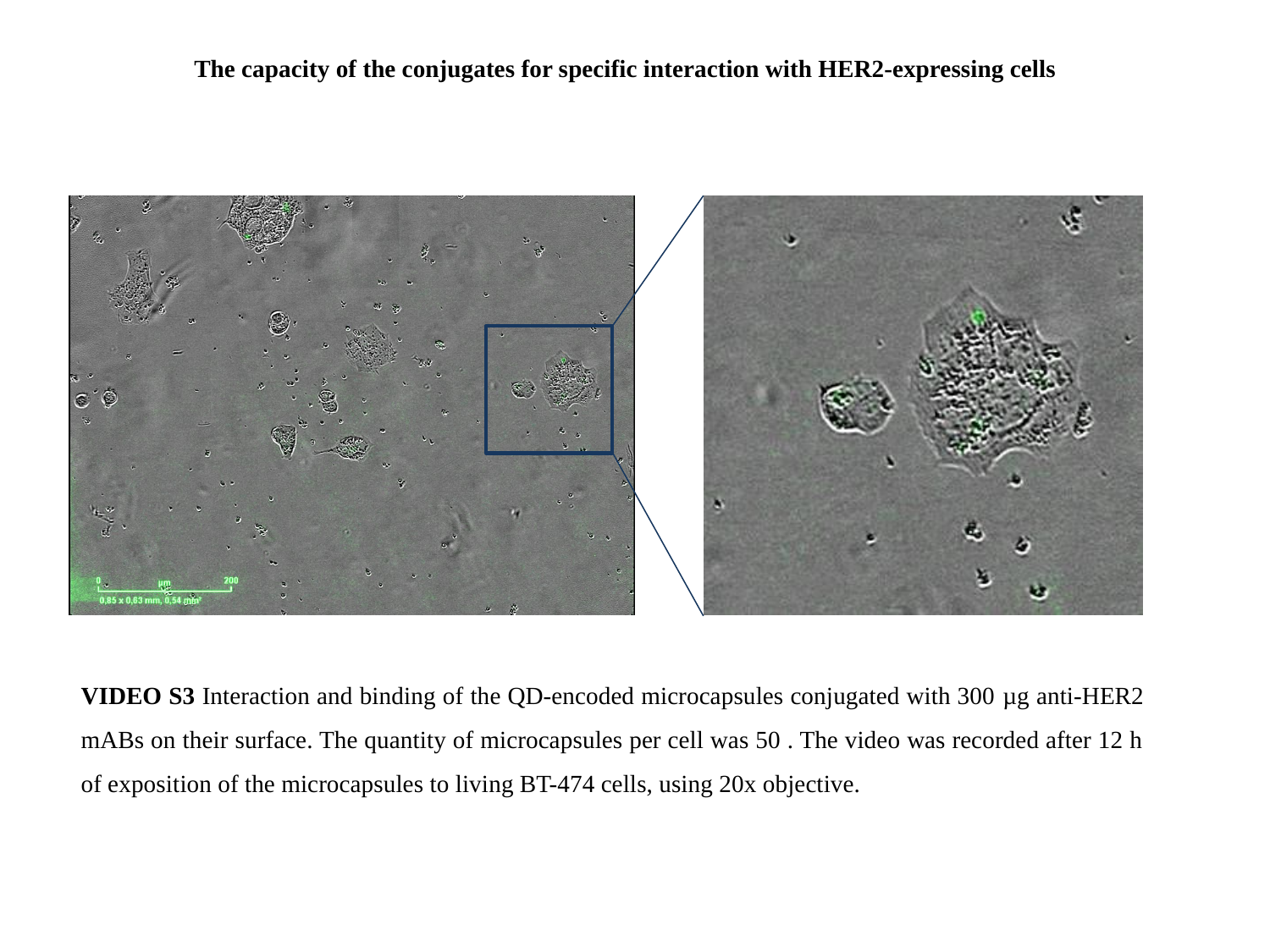

The capacity of the conjugates for specific interaction with HER2-expressing cells
VIDEO S3 Interaction and binding of the QD-encoded microcapsules conjugated with 300 µg anti-HER2 mABs on their surface. The quantity of microcapsules per cell was 50 . The video was recorded after 12 h of exposition of the microcapsules to living BT-474 cells, using 20x objective.
